# Evaluating various composite sampling modes for detecting pathogenic SARS-CoV-2 virus in raw sewage

**DOI:** 10.3389/fmicb.2023.1305967

**Published:** 2023-11-23

**Authors:** Ye Li, Kurt T. Ash, Dominique C. Joyner, Daniel E. Williams, Isabella Alamilla, Peter J. McKay, Chris Iler, Terry C. Hazen

**Affiliations:** ^1^Department of Civil and Environmental Engineering, University of Tennessee, Knoxville, Knoxville, TN, United States; ^2^Biosciences Division, Oak Ridge National Laboratory, Oak Ridge, TN, United States; ^3^Student Health Center, University of Tennessee, Knoxville, Knoxville, TN, United States; ^4^Department of Facilities Services, University of Tennessee, Knoxville, Knoxville, TN, United States; ^5^Department of Microbiology, University of Tennessee, Knoxville, Knoxville, TN, United States; ^6^Bredesen Center, University of Tennessee, Knoxville, Knoxville, TN, United States; ^7^Department of Earth and Planetary Sciences, University of Tennessee, Knoxville, Knoxville, TN, United States

**Keywords:** SARS-CoV-2 RNA, PMMoV RNA, raw sewage, sampling modes, sampling duration, sampling timing

## Abstract

Inadequate sampling approaches to wastewater analyses can introduce biases, leading to inaccurate results such as false negatives and significant over- or underestimation of average daily viral concentrations, due to the sporadic nature of viral input. To address this challenge, we conducted a field trial within the University of Tennessee residence halls, employing different composite sampling modes that encompassed different time intervals (1 h, 2 h, 4 h, 6 h, and 24 h) across various time windows (morning, afternoon, evening, and late-night). Our primary objective was to identify the optimal approach for generating representative composite samples of SARS-CoV-2 from raw wastewater. Utilizing reverse transcription-quantitative polymerase chain reaction, we quantified the levels of SARS-CoV-2 RNA and pepper mild mottle virus (PMMoV) RNA in raw sewage. Our findings consistently demonstrated that PMMoV RNA, an indicator virus of human fecal contamination in water environment, exhibited higher abundance and lower variability compared to pathogenic SARS-CoV-2 RNA. Significantly, both SARS-CoV-2 and PMMoV RNA exhibited greater variability in 1 h individual composite samples throughout the entire sampling period, contrasting with the stability observed in other time-based composite samples. Through a comprehensive analysis of various composite sampling modes using the Quade Nonparametric ANCOVA test with date, PMMoV concentration and site as covariates, we concluded that employing a composite sampler during a focused 6 h morning window for pathogenic SARS-CoV-2 RNA is a pragmatic and cost-effective strategy for achieving representative composite samples within a single day in wastewater-based epidemiology applications. This method has the potential to significantly enhance the accuracy and reliability of data collected at the community level, thereby contributing to more informed public health decision-making during a pandemic.

## 1 Introduction

Wastewater-based epidemiology (WBE) has emerged as an indispensable tool for monitoring and enhancing public health on university campuses, garnering significant global recognition. As of August 31, 2023, a recent study conducted by University of California Merced researchers reveals that 288 universities across 72 countries have adopted WBE practices (WBE Collaborative, 2023). Notably, the implementation of WBE gained substantial traction during the 2020–2021 academic year and has proven effective in reducing the prevalence of SARS-CoV-2 within university communities (Lee et al., 2023).

Sampling approaches are a crucial aspect of WBE, as improperly designed sampling approaches can lead to issues such as false negatives and substantial over- or underestimation of daily average viral concentrations. These issues stem from the inability to capture the extreme or peak values in the temporally variable viral input or the accidental acquisition of these values. On September 6, 2023, a comprehensive literature search was conducted using the Web Science Core Collection database, with no restrictions on publication date or language. The search criteria included terms associated with SARS-CoV-2, wastewater, and university campuses, yielding 154 articles on the subject. Following a thorough review of all identified papers, 108 records were eliminated due to lack of relevance to the campus, resulting in the final selection of 46 articles. Table 1 provides a summary of the information presented in these articles from 41 universities. This table demonstrates that universities employ three sampling methodologies frequently in WBE: grab sampling, composite sampling, and passive sampling. The autosampler is typically used with composite sampling. Due to the discrete and variable nature of SARS-CoV-2 inputs into a catchment, the accuracy of grab sampling is questionable despite its cost-effectiveness and efficiency compared to composite sampling. When autosamplers are inaccessible due to factors such as low flow rates, logistical restrictions, or financial constraints, grab sampling techniques are required. However, determining the precise relationship between the amount collected and the environmental concentration remains a difficult task (Wilson et al., 2022).

**Table 1:** Characteristics of included studies.

	Sampling type	Deployed time	Sampling duration	Sampling site	Target	References
1	Grab	Morning		Individual-building level	N1, E	Betancourt et al. (2021), Schmitz et al. (2021), and Vo et al. (2022)
2	Grab	Morning		Individual-building level	N1, N2 PMMoV	Scott et al. (2021)
3	Grab	Morning		Multiple-building level		Swift et al. (2022)
4	Grab			Multiple-building level	N1, N2 PMMoV	Conway et al. (2023)
5	Grab	Morning		Multiple-building level	N1, N2	Kazenelson et al. (2023)
6	Composite Grab	Morning	24 h	Individual-building level	N1, N3	Ash et al. (2023) and Li et al. (2023)
7	Composite Grab		48-96 h 48-72 h 24-30 h	Multiple-building level Individual-building level	N1 BRSV	Wartell et al. (2022)
8	Composite Grab	Morning	24 h	Campus level Multiple-building level	N1, N2, E PMMoV	Lu et al. (2022)
9	Composite Grab		24 h	Multiple-building level Individual-building level	N1, N2, PMMoV	Fahrenfeld et al. (2022)
10	Composite Grab		24 h	Campus level Multiple-building level	N1, N2 PMMoV	Bitter et al. (2022)
11	Composite Grab	Morning	24 h	Individual-building level	N1, N2	Rainey et al. (2022)
12	Composite Grab	Morning	24 h	Individual-building level Multiple-building level	N1, N2, N3 PMMoV, B2M, fecal coliform	Sharkey et al. (2021), Zhan et al. (2022), and Solo-Gabriele et al. (2023)
13	Composite Grab			Individual-building level	N1, N2	Swain et al. (2023)
14	Passive Grab	Morning	24-48 h 72 h	Campus level Individual-building level	N1 BRSV	Liu et al. (2022) and Wang et al. (2022)
15	Composite		24 h	Campus level Individual-building level	N1	Sweetapple et al. (2022)
16	Composite	Morning	24 h		N1, N2 PMMoV	Sellers et al. (2022)
17	Composite	Morning	20-24 h 20-22 h	Multiple-building level	N1, N2	Colosi et al. (2021) and Kotay et al. (2022)
18	Composite	Morning	24 h 12 h 3 h	Multiple-building level	N1, N2	Anderson-Coughlin et al. (2022)
19	Composite	Morning	24 h	Multiple-building level	E	Wright et al. (2022)
20	Composite		24 h	Individual-building level	N1 BCoV PMMoV	Welling et al. (2022)
21	Composite	Morning	6 h 12 h	Multiple-building level	N1 PMMoV	Mohapatra et al. (2023)
22	Composite			Campus level Multiple-building level Individual-building level.	N1, N2, PMMoV	Zambrana et al. (2022)
23	Composite	Morning.	24 h	Individual-building level	N1, N2, E BCoV Genogroup II F+	Reeves et al. (2021) and Johnson et al. (2022)
24	Composite		24 h	Multiple-building level Individual-building level WWTP	N1, N2 BCoV PMMoV	Lee et al. (2023)
25	Composite	Morning	24 h	Individual-building level	N1, N2 BCoV PMMoV	Langan et al. (2022)
26	Composite	Morning	4-24 h	Multiple-building level	N1	Strike et al. (2022)
27	Composite	Morning	24 h 12 h	Individual-building level	N1	Rondeau et al. (2023)
28	Composite	Morning.	8.5 h	Multiple-building level Individual-building level	N1, E	de Llanos et al. (2022)
29	Composite	Morning	24 h	Individual-building level Multiple-building level	N1	Gibas et al. (2021)
30	Composite		24 h	Multiple-building level	N1, N2, E	Karthikeyan et al. (2021, 2022)
31	Composite	Morning	24 h	Campus level	N1, N2 PMMoV	Lee et al. (2022)
32	Composite	Morning	48-72 h 24-30 h	Multiple-building level Individual-building level	N1 BRSV	Wartell et al. (2022)
33	Composite	Morning	16 h	Multiple-building level	E	Sharaby et al. (2023)
34	Composite		24 h	Multiple-building level	N1	Chua et al. (2023)
35	Composite Passive	Morning	24 h	Multiple-building level Campus level	N1 BRSV	Bivins and Bibby (2021), Bivins et al. (2021), and Cavany et al. (2022)
36	Composite Grab Passive	Morning	72-96 h	Individual-building level	N1, N2 PMMoV	Cha et al. (2023)
37	Passive	Afternoon	21 h	Multiple-building level	N1, N2	Mangwana et al. (2022)
38	Passive	Morning	24 h 72 h	Individual-building level		Jain et al. (2022)
39	Passive		24 h	Individual-building level	N1, N2	Acer et al. (2022)
40	Passive		48-72 h	Multiple-building level	N1	Yaglom et al. (2022)
41	Passive		24 h	Individual-building level	N1, N2 PMMoV	Corchis-Scott et al. (2023)

N1, N2, and E, SARS-CoV-2 gene-targets; PMMoV, Pepper mild mottle virus; BCoV, Bovine Coronavirus; BRSV, Bovine Respiratory Syncytial Virus; B2M, Human-specific general house-keeping gene.

Composite sampling methods are frequently favored in universities for the purpose of wastewater sampling, as seen by the data presented in Table 1. Indeed, a majority of 30 out of the total of 41 colleges opted to employ a composite sampling approach. The temporal extent during which samples are gathered is a critical determinant in composite sampling. A significant proportion of colleges have implemented a standardized sampling time of 24 h, but other durations ranging from 3 to 96 h have been used by some institutions. Although it is commonly believed that increasing the period of composite sampling results in more representative samples, Ahmed et al. (2021) give an alternative viewpoint. The authors suggest that employing shorter sampling intervals, such as 2 h, 4 h, and 6 h, within a 24 h timeframe, could potentially produce results that are more representative. This is particularly relevant in scenarios where the distance between buildings and sampling sites is comparatively short. Furthermore, the temporal aspect of sampling emerges as an additional crucial factor to be considered when dealing with shorter sampling durations. In a recent study conducted by Randazzo et al. (2020), it has been proposed that the collection of wastewater samples in the morning may be a potentially effective approach for the surveillance of SARS-CoV-2. This hypothesis is based on the observation that the morning period coincides with a time of an increase in human activity, which could potentially result in increased viral shedding from individuals and, consequently, higher viral loads in the collected samples. The aforementioned observation potentially provides a rationale for the prevalent practice among educational institutions to initiate their sample collection protocols in the early hours of the day, as indicated by the data presented in Table 1. However, the sampling process used by Jain et al. (2022) started in the afternoon and lasted for a total of 21 h, resulting in the omission of a segment of the morning wastewater. In order to assess the reliability of the sampling times (morning, afternoon, evening, and late night) in depicting the COVID-19 trend, a thorough investigation is necessary.

To address these challenges, we undertook a comprehensive comparative analysis of various sampling durations (1 h, 2 h, 4 h, 6 h, and 24 h) and sampling times (morning, afternoon, evening, and late night) using autosamplers to collect raw sewage. Our objective was to identify the most effective strategy for achieving representative sampling of the pathogenic virus SARS-CoV-2. Given that PMMoV is a widely used indicator virus for normalizing SARS-CoV-2 data (as shown in Table 1), it is also crucial to assess the abundance and variability of PMMoV in comparison to SARS-CoV-2 across different composite sampling durations. This assessment has multiple benefits, such as, improving result accuracy, increasing data collection efficiency, reducing labor needs, optimizing laboratory resource utilization, and addressing issues like autosampler battery depletion and viral decay.

## 2 Materials and methods

### 2.1 Sewage sampling

Wastewater was collected from 3 buildings in the University of Tennessee, Knoxville campus. These residence halls accommodated more than 400 students. Two Hach AS950 Portable Peristaltic Samplers (Hach, Loveland, CO) were used to collect up to 24 discrete samples from September 14, 2020, to September 21, 2021. To procure these samples from specific dormitories with predefined student populations, the collection process involved sampling from the downstream of dispense valves or sewer manholes before their contents were mixed with other sewer lines. The characteristics of these dormitories are summarized in Table 2. The autosampler was programmed to collect a 300 or 400-milliliter sample every hour over a 24 h duration. However, a few samples were not collected due to low flow rates or batteries that were out of power. The autosamplers were promptly transported to the BSL-2 laboratory on campus the following day within 3 h of the final collection interval.

**Table 2:** Characteristics of different time-based composite samples.

Time-based composite sample	Sampling site	Sampling point	Sampling date	Student number	pH
1 h	D1	Manhole	Nov 12 2020	595	NA
			Dec 01 2020	595	NA
			Dec 17 2020	595	NA
			Jan 04 2021	590	7.30–8.70
2 h	D1	Manhole	Dec 28 2020	595	NA
			Feb 08 2021	590	7.64–8.78
			Feb 1,520,221	590	6.90–8.78
4 h	D1	Manhole	Jan 11 2021	590	7.60–8.60
			Jan 25 2021	590	7.67–8.78
			Feb 01 2021	590	NA
			Mar 02 2021	590	7.69–8.53
			Mar 08 2021	590	7.31–8.98
			Mar 15 2021	590	7.67–8.64
			Mar 22 2021	590	7.34–8.49
	D2	Direct Dispense from valve	Jan 20 2021	580	NA
			Jan 26 2021	580	NA
6 h	D1	Manhole	Dec 09 2020	595	NA
			Jan 19 2021	590	8.03–8.62
	D3	Manhole	Jan 192,021	454	7.76–7.97

### 2.2 Sample processing

**Figure 1:**
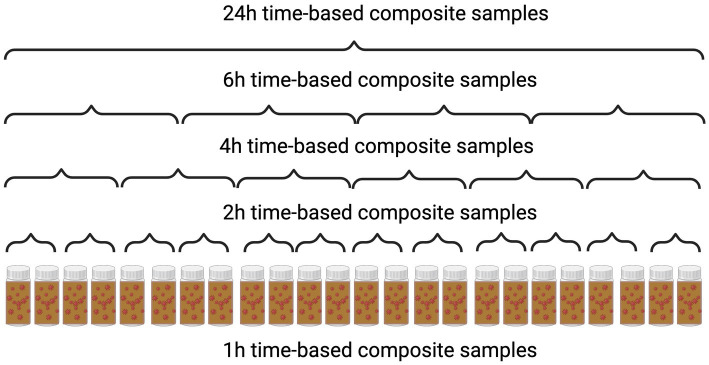
Sampling process diagram of different time-based composite samples.

Upon arrival at the laboratory, the composite samples were manually mixed thoroughly and uniformly to incorporate time points at 2, 4, 6, or 24 h within the 24 h period into a 500 mL bottle (Figure 1). Furthermore, we opted to choose multiple composite samples of 2 h and 4 h durations, representing the different sampling times (morning, afternoon, night) from a single 24 h time period. We then divided an equal volume from each of these samples and combined them to form a single composite sample representing the entire 24 h period.

The sewage samples were processed through a series of steps: they were initially pasteurized for 2 h at 60°C in a water bath, followed by centrifugation at 5,000 × g for 10 min. To remove any substantial suspended particulate matter, the samples were then filtered through nitrocellulose filters with pore sizes of 0.45 μm and 0.22 μm. Subsequently, concentration was achieved using an Amicon Ultra-15 filtration device, with centrifugation at either 4,000 × g for 30 min (Swing-arm rotor) or 5,000 × g for 20 min (Fixed-angle rotor) at room temperature. The resulting concentrated solution, approximately 250 μL, was carefully transferred to 2 mL DNA LoBind tubes. RNA extraction was performed using the Qiagen viral RNA Mini Kit, following manufacturer instructions, yielding 60 μL of extracted RNA, with a negative control using DNase/RNase-free water. Finally, the RNA samples were stored at −80°C and subsequently subjected to RT-qPCR analysis within 24 h following extraction (Ash et al., 2023; Li et al., 2023).

### 2.3 RT-qPCR

To quantify the concentrations of SARS-CoV-2 and PMMoV RNA in each sample, we utilized RT-qPCR. Specifically, we quantified SARS-CoV-2 N1 using the TaqPath 1-Step RT-qPCR Master Mix, CG (Thermo Fisher Scientific) on an Applied Biosystems QuantStudios 7 Pro Real-Time PCR System instrument. Each 20 μL reaction mixture consisted of 5 μL of 4X Master Mix (Thermo Fisher Scientific), 0.25 μL of a 10 μmol/L probe, 1 μL each of 10 μmol/L forward and reverse primers, 7.75 μL of nuclease-free water, and 5 μL of nucleic acid extract. Accurate pipetting of reagents into 96-well plates was followed by 10 s of vortex mixing. The RT-qPCR cycling conditions encompassed initial uracil-DNA glycosylase incubation for 2 min at 25°C, reverse transcription for 15 min at 50°C, activation of the Taq enzyme for 2 min at 95°C, and a two-step cycling process involving 3 s at 95°C and 30 s at 55°C, repeated for a total of 45 cycles. A positive test result was determined by the presence of an exponential fluorescent curve that intersected the threshold within 40 cycles (cycle threshold [Ct] <40).

The quantification of PMMoV was performed using the TaqPath 1-Step RT-qPCR Master Mix, CG (Thermo Fisher Scientific) on a QuantStudios 7 Pro instrument. Each reaction consisted of 20 μL, comprising 5 μL of 4X Master Mix from Thermo Fisher Scientific, 0.5 μL of 10 μmol/L probe, 1.8 μL each of 10 μmol/L forward and reverse primers, 8.9 μL of nuclease-free water, and 2 μL of nucleic acid extract. The reagents were carefully transferred into 96-well plates using pipettes and subsequently mixed by vortexing for 10 s. The thermocycling conditions utilized in this study were as follows: incubation of uracil-DNA glycosylase for 2 min at 25°C, reverse transcription carried out for 15 min at 50°C, activation of the Taq enzyme for 10 min at 95°C, and a two-step cycling process consisting of 30 s at 95°C followed by 1 min at 60°C, repeated for a total of 40 cycles.

Each RT-qPCR run included one positive PMMoV controls and negative controls, consisting of Mastermix and DNase/RNase-free water. The RT-qPCR reactions were conducted in triplicate. The criterion for classifying a sample as positive was that all replicates yielded positive results, with each individual replicate falling within the linear range of the standard curve. The N1 standard curve exhibited a high level of efficiency, with a value of 94.669% (*R*^2^ = 1). The quantification of SARS-CoV-2 RNA was determined by calculating the average of three replicates of viral copies. The outputs of RT-qPCR were transformed into units of copies per liter. In this study, the detection limit for SARS-CoV-2 and PMMoV was determined to be 20 and 10 copies per liter, respectively.

### 2.4 Data analysis

All statistical analyses were performed using SPSS (IBM Corp. Released 2019. IBM SPSS Statistics for Windows, Version 26.0. Armonk, NY: IBM Corp). The data were examined for normality and homogeneity of variance. The paired *t*-test was used to compare 24 h average and 24 h time-based composite samples of SARS-CoV-2 and PMMoV RNA. Due to the non-normal distribution of the data in our study, the Quade Nonparametric ANCOVA test was performed on composite samples throughout a range of sampling durations (1 h, 2 h, 4 h, 6 h, and 24 h) and sampling times (morning, afternoon, evening, and late-night), with the date and PMMoV concentration as covariates. All statistical differences were determined by *p* < 0.01.

## 3 Results

### 3.1 Concentration and variability of SARS-CoV-2 and PMMoV in raw sewage

Table 3 displays the pathogenic virus of SARS-CoV-2 and indicator virus of PMMoV RNA concentrations of 24 h average and 24 h composite samples collected at 2 h and 4 h time intervals. The 24 h average composite sample is the numeric average concentration of the 24 h period samples. Comparing the 24 h average concentrations with the corresponding 24 h composite samples for both SARS-CoV-2 and PMMoV, no significant differences were observed. However, there were significant positive correlations between the 24 h average concentrations and the corresponding 24 h composite samples for both SARS-CoV-2 and PMMoV (paired *t*-test, Correlation = 0.985 and 0.989, *value of p* = 0.002 and 0.001, for SARS-CoV-2 and PMMoV).

**Table 3:** Concentration of SARS-CoV-2 and PMMoV RNA in 2 h, 24 h average, and 24 h time-based composite samples, and 4 h, 24 h average, and 24 h time-based composite samples.

Virus	2 h (Log_10_ Copies/L)			4 h (Log_10_ Copies/L)			Paired *t*	*P*
	Sample	24 h average	24 h composite	Sample	24 h average	24 h composite		
SARS-CoV-2	A	1.60	1.75	C	2.29	2.47	1.898	0.131
	B	2.19	2.40	D	3.74	3.81		
				E	2.59	3.20		
PMMoV	A	4.50	4.42	C	4.08	4.12	−0.716	0.513
	B	3.64	3.48	D	3.67	3.74		
				E	3.50	3.56		

**Figure 2:**
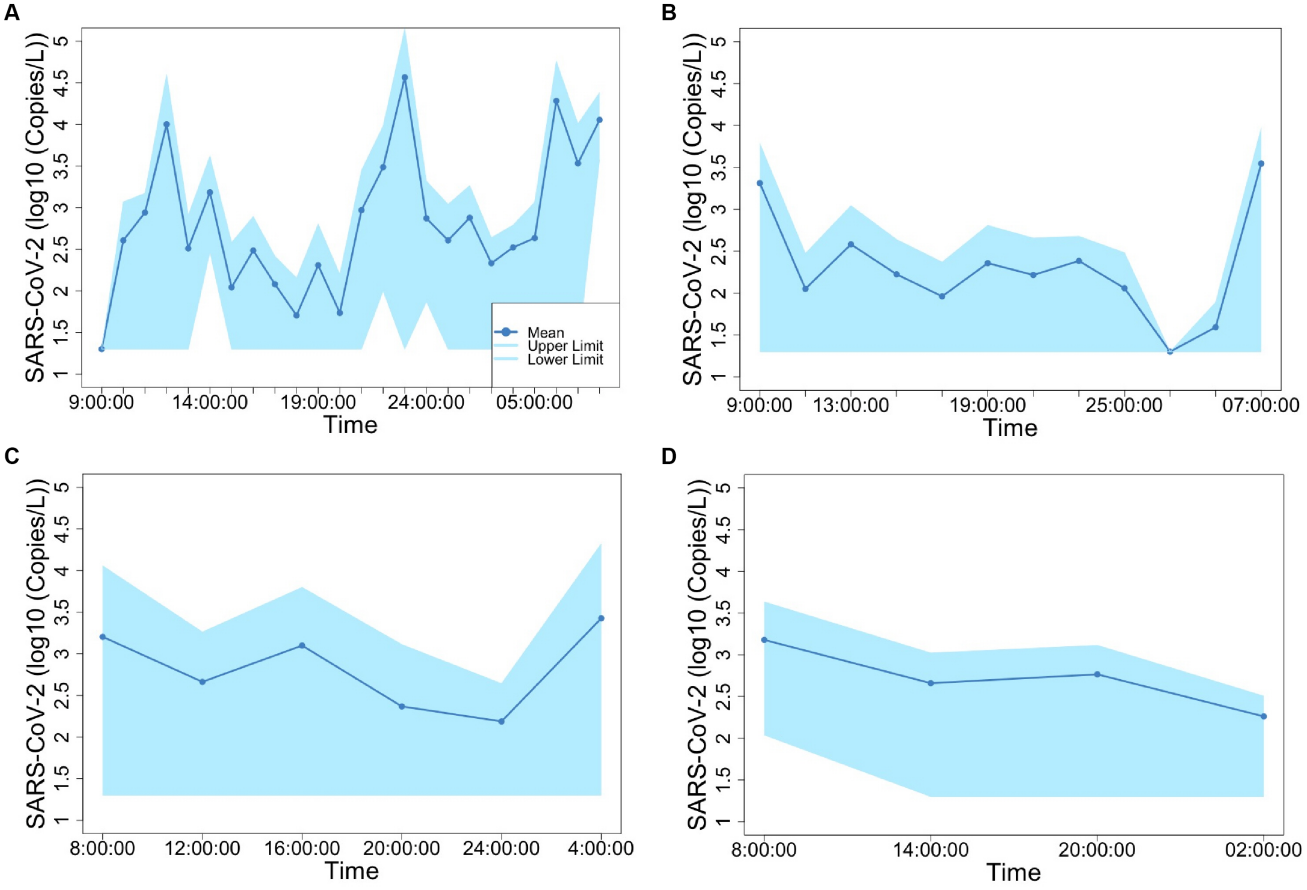
Concentration of SARS-CoV-2 fluctuation in 1-h **(A)**, 2-h **(B)**, 4-h **(C)**, and 6-h **(D)** time-based composite samples.

**Figure 3:**
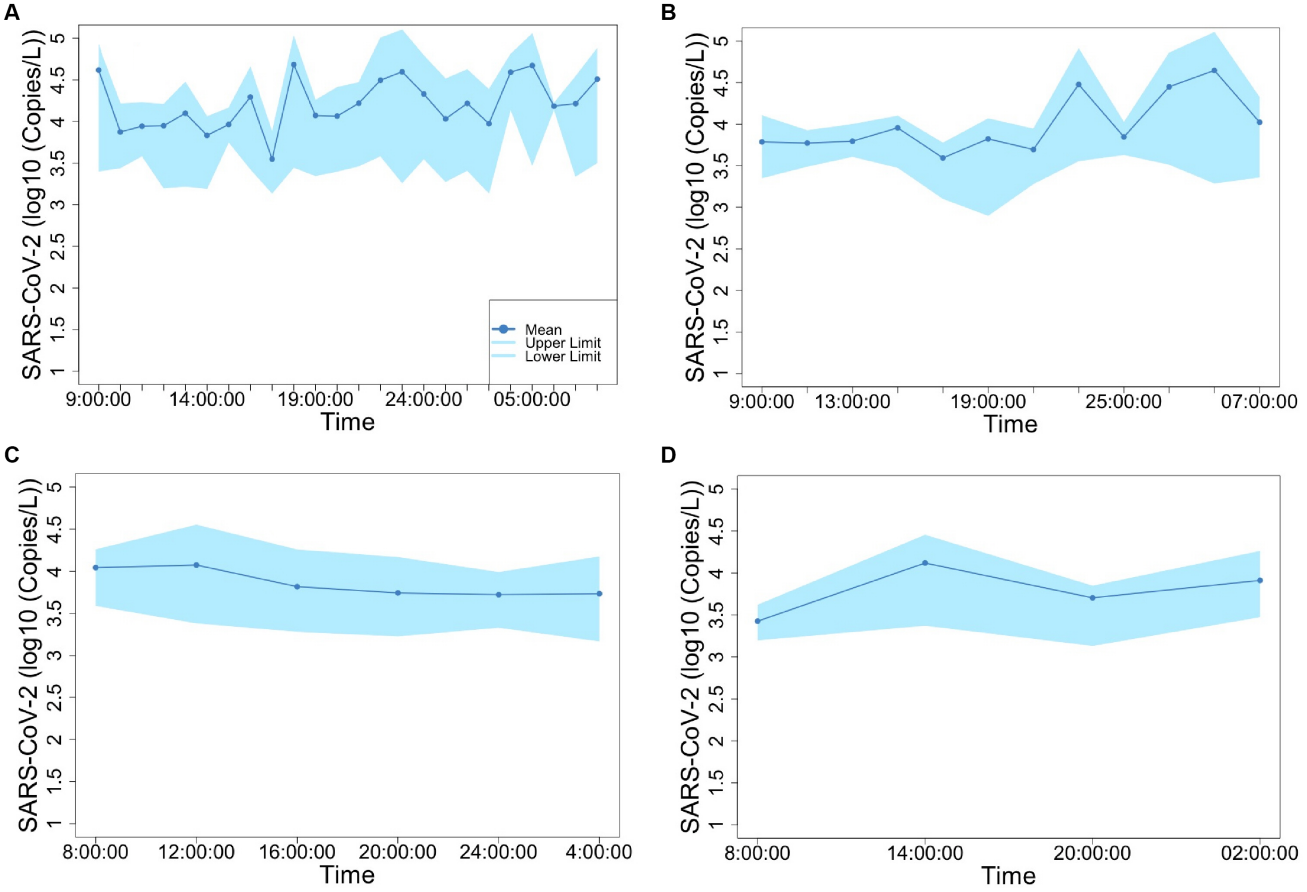
Concentration of PMMoV fluctuation in 1-h **(A)**, 2-h **(B)**, 4-h **(C)**, and 6-h **(D)** time-based composite samples.

**Figure 4:**
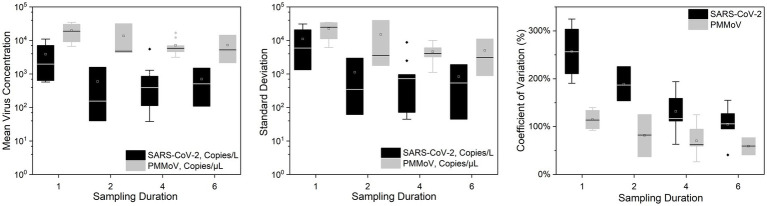
Mean virus concentration, standard deviations, and coefficient of variation of SARS-CoV-2 and PMMoV RNA on different sampling durations of composite samples.

Figures 2, 3 show the concentrations of SARS-CoV-2 and PMMoV RNA in composite samples collected at different time intervals (1 h, 2 h, 4 h, and 6 h). During the sampling period, the concentration of SARS-CoV-2 RNA was 3.60 ± 4.24, 2.77 ± 3.26, 3.06 ± 3.54, 2.83 ± 3.08, 3.22 ± 3.45 log10 copies/L in the 1 h, 2 h, 4 h, 6 h, and 24 h time-based composite samples, respectively. Meanwhile, the corresponding concentrations of PMMoV RNA were 4.2 ± 4.43, 4.13 ± 4.41, 3.87 ± 3.81, 3.86 ± 3.91, 4.06 ± 4.02 log10 copies/L in the 1 h, 2 h, 4 h, 6 h, and 24 h time-based composite samples, respectively. The mean concentration of PMMoV RNA was significantly greater than the mean concentrations of SARS-CoV-2 RNA in all the time-based composite samples, respectively (paired *t*-test, *t* = −3.884, value of *p* <0.01, Figure 4). The standard deviations (SDs) of SARS-CoV-2 and PMMoV RNA were calculated to assess the variation within each time-based composite sample. Notably, the variation of SARS-CoV-2 did not show significant differences with the PMMoV RNA in 1 h, 2 h, 4 h, 6 h, and 24 h time-based composite samples, respectively (Figure 4). The coefficient of variation values (CVs) were calculated to further assess the variation within each time-based composite sample for SARS-CoV-2 and PMMoV RNA. For SARS-CoV-2 RNA, the CVs ranged from 1.91 to 0.33, 1.54 to 2.25, 0.63 to 1.94, and 1.06 to 1.27 in the 1 h, 2 h, 4 h, and 6 h time-based composite samples, respectively. In contrast, the CVs for PMMoV RNA were significantly smaller than those of SARS-CoV-2 RNA (paired *t*-test, *t* = 5.150, value of *p* <0.01), ranging from 0.92 to 1.40, 0.37 to 1.25, 0.27, to 1.25, and 0.41 to 0.77 in the 1 h, 2 h, 4 h, and 6 h time-based composite samples, respectively (Figure 4).

### 3.2 The impact of different sampling durations and time periods on composite samples

**Figure 5:**
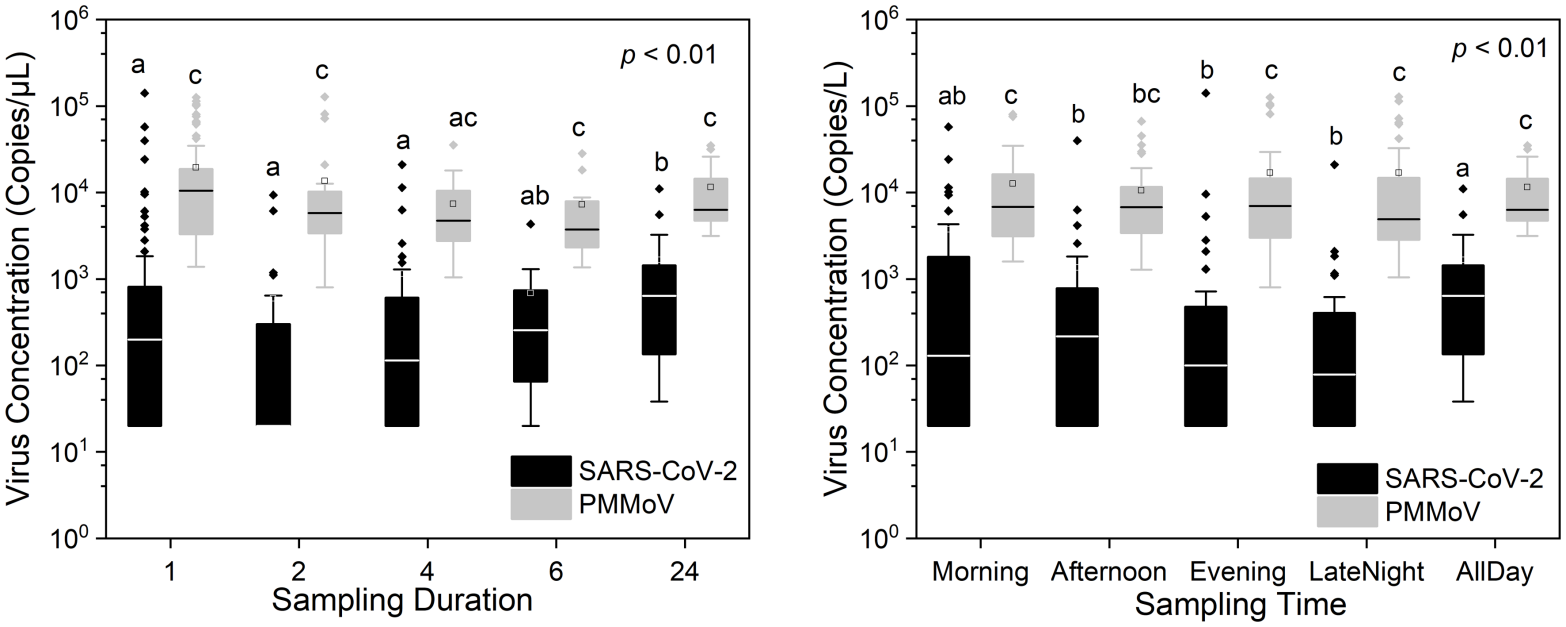
Different sampling durations and time periods of composite samples.

Figure 5 illustrates the different sampling durations and time periods used for composite samples in this study. As discussed earlier, the 24 h average concentrations of SARS-CoV-2 and PMMoV RNA accurately represent the 24 h composite samples within their respective sampling intervals. So the 24 h composite samples in this study were derived from the 24 h average concentrations of SARS-CoV-2 and PMMoV RNA. Statistical analysis using the Quade Nonparametric ANCOVA test revealed a significant difference in SARS-CoV-2 RNA concentrations between the 24 h time-based composite samples and the 1 h, 2 h, and 4 h time-based composite samples (*t* = −3.066, −4.311, −2.790, respectively, *p* < 0.01), with date, concentration of PMMoV and sampling sites as covariates. However, there was no significant difference observed in SARS-CoV-2 RNA concentrations between the 24 h and 6 h time-based composite samples. In addition, there was no significant difference observed in PMMoV RNA concentrations between the 24 h composite samples and other time-based composite samples with date as covariates.

In addition, our statistical analysis, utilizing the Quade Nonparametric ANCOVA test with date and concentration of PMMoV and sampling sites as covariates, the morning time period showed no significance with the 24 h composite samples compared to other time periods (*t* = −2.388, *p* > 0.01, Figure 5). The morning time frame is defined as sampling from 6:01 am to 12:00 pm, the afternoon from 12:01 pm to 6:00 pm, the evening from 6:01 pm to 12:00 am, and the late-night hours from 12:01 am to 6:00 am. However, there were no significant variations observed in the concentrations of PMMoV RNA among the various time periods. These findings highlight the potential importance of selecting the morning time for sampling the pathogenic virus, as it could provide a representative snapshot of the entire day’s virus concentration levels. This information can be valuable for designing sampling strategies aimed at capturing accurate and comprehensive data on SARS-CoV-2 RNA concentrations throughout the day.

## 4 Discussion

Our findings indicate that the 24 h average concentrations of both SARS-CoV-2 and PMMoV RNA are consistent with the corresponding 24 h composite samples. This suggests that the 24 h average concentrations of SARS-CoV-2 and PMMoV RNA could accurately represent the 24 h composite samples over the respective sampling intervals.

Our results reveal a consistent pattern where the mean concentration of PMMoV RNA exceeded that of SARS-CoV-2 RNA across all time-based composite samples These results indicate that, on average, the abundance of PMMoV RNA was higher than that of SARS-CoV-2 RNA in the samples obtained at various time intervals. The difference in prevalence between PMMoV and SARS-CoV-2 can be attributed to the differential shedding patterns and origins of these viruses. PMMoV, being an indicator virus commonly used for detection purposes, is excreted by both asymptomatic and symptomatic individuals, with dietary consumption playing a role in its transmission (Rosario et al., 2009; Wu et al., 2020). On the other hand, SARS-CoV-2 is a virulent virus primarily released by individuals who have contracted the infection, resulting in a narrower source of transmission and lower prevalence compared to PMMoV.

Both SARS-CoV-2 and PMMoV RNA exhibited greater variability in the 1 h individual composite samples compared to longer time-based composite samples highlighting an important consideration in wastewater surveillance. The present investigation aligns with the research conducted by Bertanza et al. (2022), as it demonstrates that applying shorter sampling intervals, such as the 1 h interval employed in this study, facilitates a more comprehensive view of the temporal fluctuations in substance dynamics within a 24 h period. It is crucial to acknowledge that the SDs of PMMoV, used as an indicator virus, did not exhibit a significant difference from the pathogenic virus SARS-CoV-2. This implies that there is no statistically significant distinction between these two viruses when assessing variation using SDs. Furthermore, the CVs also offer valuable insights into the variation and consistency of viral RNA concentrations within different time-based composite samples of wastewater. The finding that CVs for SARS-CoV-2 RNA were notably higher compared to those of PMMoV RNA underscores the contrasting dynamics of these viruses in wastewater. The lower CVs for PMMoV RNA indicate that the concentrations of PMMoV RNA in each time-based composite sample were more consistent and less variable compared to the concentrations of SARS-CoV-2 RNA (Wu et al., 2022), particularly in the 1 h and 2 h composite samples (Figure 5). This is likely because the concentrations of PMMoV RNA in a sewage system depend directly on the diet of infected pepper plants by the contributing populations (Ash et al., 2023), which may vary throughout the day, leading to fluctuating CVs. On the other hand, pathogenic viruses like SARS-CoV-2 or other enteric viruses, which are typically present in lower concentrations, are expected to exhibit more variability throughout the day. This observation is consistent with the results reported by Ahmed et al. (2021), which demonstrated that the concentrations of indicator viruses of crAssphage and PMMoV exhibited significantly lower variability compared to the pathogenic virus HAdV.

The results of this study shed light on the selection of appropriate sampling modes for studying the temporal variations in SARS-CoV-2 and PMMoV RNA concentrations. The significant difference in SARS-CoV-2 RNA concentrations between the 24 h time-based composite samples and shorter time intervals (1 h, 2 h, and 4 h) highlights the dynamic nature of SARS-CoV-2 RNA concentration patterns over shorter time frames. This suggests that shorter composite intervals are essential for capturing rapid fluctuations in SARS-CoV-2 RNA concentrations, which could be crucial for monitoring and responding to outbreaks in real-time. The lack of a significant difference between the 24 h and 6 h time-based composite samples for SARS-CoV-2 RNA suggests that a 6 h sampling interval can effectively capture the overall trends and patterns in SARS-CoV-2 RNA concentrations over a 24 h period. This finding has practical implications, as it allows for a more efficient sampling strategy while maintaining accuracy. Furthermore, the study’s comparison to previous research, such as Mohapatra et al. (2023) and Wartell et al. (2022), indicates that different sampling intervals may be suitable depending on the specific objectives of a study. For instance, transitioning from 6 h to 12 h composites, as observed by Mohapatra et al. (2023), did not significantly impact sensitivity in their detection methods, suggesting flexibility in designing sampling protocols. In contrast, the lack of significant differences in PMMoV RNA concentrations between various time-based composite samples implies that PMMoV RNA exhibits less temporal variability within the studied time frames. This information is essential for understanding the behavior of PMMoV RNA and selecting appropriate sampling strategies when studying this particular virus.

The results of our statistical analysis demonstrate a significant temporal variation in SARS-CoV-2 RNA concentrations, which is influenced by the time of day at which samples were collected. This finding underscores the importance of considering diurnal patterns when designing sampling strategies for monitoring viral presence and concentration in a given environment. The distinct differences observed between the morning and other time periods in terms of SARS-CoV-2 RNA concentrations are particularly noteworthy. The reduced variability in the morning samples with the 24 h samples suggests that this time frame may be more suitable for accurately assessing the daily viral dynamics within the sampled environment. The morning period corresponds to a time when human activity typically surges, potentially leading to increased viral shedding from individuals and subsequently higher viral loads in the collected samples. This is consistent with most universities that they initiated their sample collection protocols in the early hours of the day, as indicated in Table 1. Researchers and public health officials should take this into account when planning surveillance and sampling efforts. Understanding these temporal variations in viral concentrations can have practical implications for public health strategies, especially in settings like campuses, where individuals congregate and interact. It highlights the importance of targeted sampling during peak activity periods to ensure that monitoring efforts accurately reflect the potential viral risk within a community. The lack of significant variations in PMMoV RNA concentrations across the different time periods indicates that PMMoV may not exhibit the same diurnal patterns as SARS-CoV-2. This finding is valuable in understanding the behavior of PMMoV and suggests that its dynamics may be less influenced by daily fluctuations.

## 5 Conclusion

This study conducted a field trial to assess various sampling modes and timings using autosamplers for the detection of the pathogenic virus SARS-CoV-2 and the indicator virus PMMoV RNA in raw sewage collected from university dormitories. The findings highlight the efficacy of 24 h composite samples in reducing variability, particularly when targeting pathogenic viruses such as SARS-CoV-2. Furthermore, our research also suggests that employing a composite sampler during a focused 6 h morning window can provide a practical, cost-effective, and time-efficient method to ensure representative sampling in wastewater-based epidemiology applications. This insight contributes valuable options for optimizing wastewater sampling strategies in the context of pathogen detection and epidemiological surveillance.

## Data availability statement

The original contributions presented in the study are included in the article/supplementary material, further inquiries can be directed to the corresponding author.

## Author contributions

YL: Data curation, Formal analysis, Investigation, Methodology, Validation, Visualization, Writing – original draft, Writing – review & editing. KA: Formal analysis, Investigation, Methodology, Resources, Validation, Writing – review & editing. DJ: Investigation, Methodology, Validation, Writing – review & editing. DW: Investigation, Methodology, Validation, Writing – review & editing. IA: Investigation, Methodology, Writing – review & editing. PM: Investigation, Methodology, Writing – review & editing. CI: Investigation, Methodology, Project administration, Writing – review & editing. TH: Funding acquisition, Project administration, Supervision, Writing – original draft, Writing – review & editing.
